# High Prevalence of Rotavirus A in Raw Sewage Samples from Northeast Spain

**DOI:** 10.3390/v12030318

**Published:** 2020-03-16

**Authors:** Marcelle Silva-Sales, Sandra Martínez-Puchol, Eloy Gonzales-Gustavson, Ayalkibet Hundesa, Rosina Gironès

**Affiliations:** Microbiology, Virology and Biotechnology Section, Department of Genetics, Microbiology and Statistics, Faculty of Biology, University of Barcelona, Av. Diagonal 643, Barcelona 08028, Spain; smpuchol@gmail.com (S.M.-P.); gonzaleseloy@gmail.com (E.G.-G.); ahundesa@ub.edu (A.H.); rgirones@ub.edu (R.G.)

**Keywords:** rotavirus A, wastewater, viral quantification, molecular characterization

## Abstract

Rotavirus A (RVA) is the most common virus associated with infantile gastroenteritis worldwide, being a public health threat, as it is excreted in large amounts in stool and can persist in the environment for extended periods. In this study, we performed the detection of RVA and human adenovirus (HAdV) by TaqMan qPCR and assessed the circulation of RVA genotypes in three wastewater treatment plants (WWTPs) between 2015 and 2016 in Catalonia, Spain. RVA was detected in 90% and HAdV in 100% of the WWTP samples, with viral loads ranging between 3.96 × 10^4^ and 3.30 × 10^8^ RT-PCR Units/L and 9.51 × 10^4^ and 1.16 × 10^6^ genomic copies/L, respectively. RVA VP7 and VP4 gene analysis revealed the circulation of G2, G3, G9, G12, P[4], P[8], P[9] and P[10]. Nucleotide sequencing (VP6 fragment) showed the circulation of I1 and I2 genotypes, commonly associated with human, bovine and porcine strains. It is important to mention that the RVA strains isolated from the WWTPs were different from those recovered from piglets and calves living in the same area of single sampling in 2016. These data highlight the importance of monitoring water matrices for RVA epidemiology and may be a useful tool to evaluate and predict possible emergence/reemergence of uncommon strains in a region.

## 1. Introduction

Rotavirus A (RVA) is the most common pathogen associated with acute gastroenteritis in children worldwide. It accounts for approximately 215,000 deaths annually of children up to 5 years old [1]. In Europe, RVA is responsible for 75,000–150,000 infantile hospitalization due to acute gastroenteritis associated with RVA infections. In Spain, the annual incidence of acute gastroenteritis associated with RVA in primary care ranges between 15.4 and 19.5 cases per 1000 children up to 5 years and 20 cases per 1000 children up to 3 years [2].

RVA is one of the nine groups (A–I) of rotavirus established according to the International Committee on Taxonomy of Viruses (ICTV) (ICTV, updated in July 2018 and available at https://talk.ictvonline.org/taxonomy/). The virion is non-enveloped, icosahedral, with a triple-layered protein capsid and consists of 11 segments of double-stranded genomic RNA (dsRNA), which encode six structural (VP1–4, 6 and 7) and six non-structural (NSP1–6) proteins. The binary classification system for RVA is based on molecular analysis of the two outer capsid proteins, VP7 and VP4, which define the G (for glycoprotein) and P (for protease sensitive) genotypes, respectively. To date, there are 36 G genotypes and 51 P genotypes described in the literature (Rotavirus Classification Working Group, updated in May 2018 and available at https://rega.kuleuven.be/cev/viralmetagenomics/virus-classification/rcwg). Six G/P combinations: G1P[8], G2P[4], G3P[8], G4P[8], G9P[8] and G12P[8] are the most prevalent combinations detected in humans [3,4,5,6,7,8]. 

However, RVA genotypes derived from animals have also been identified in humans, demonstrating that animals can act as a source of infection for humans. It is believed that animal RVA that crosses the interspecies barrier is not able to infect or disseminate efficiently in the new host. However, if such strains acquired gene segments of human origin, this would increase the chance of efficiently infecting humans [9].

With an average population of 150 million pigs, the European Union is the world’s second largest producer of pig meat (with the countries that produce the most being France, Germany and Spain) after China; it is also the largest exporter of pig meat and pig meat products (accessed at https://ec.europa.eu/agriculture/pigmeat_en, on 14th October 2018). The Spanish region of Catalonia is responsible for 44% of all pig meat produced in the country; its production is greater than that of Italy, Belgium and the Netherlands, and it exports more than Brazil, France or Poland (accessed at http://generalitatgirona.gencat.cat/ca/detalls/Noticia/Nova-Noticia-04179, on 30th January 2017).

RVA is excreted at high concentration in stools from infected humans and animals (1 × 10^11^ virus particles per gram of stool), is very stable and resistant to different methods of wastewater treatments, thus it can persist in the environment for extended periods serving as a source of infection for susceptible humans and animals [10,11].

Vaccination is considered the most efficient strategy to reduce the impact of RVA infection. Since 2006, two anti-RVA vaccines have been licensed worldwide, Rotarix^®^ and Rotateq^®^. Rotarix^®^ (RV1) (GlaxoSmithKline, Rixensart, Belgium) is an attenuated monovalent vaccine, derived from a human G1P[8] strain, which is administered orally to infants at 2 and 4 months of age. Rotateq^®^ (RV5) (Merck and Co., Whitehouse Station, NJ, USA; Sanofi Pasteur MSD, Lyon, France) is a pentavalent vaccine comprising five human-bovine reassortant strains: four expressing human G1, G2, G3, G4 and bovine P7[5], and one expressing P1A[8] (human) and G6 (bovine). This latter vaccine is administered orally to infants at 2, 4 and 6 months of age [12]. Both vaccines were licensed in Spain in late 2006, and although the Advisory Board on Vaccines of the Spanish Association of Pediatricians began to recommend vaccination against RVA in all eligible children in 2008, RV1 and RV5 are not funded by the public health system, being only available in the private market [13].

This study aims to increase the availability of information concerning the circulation of RVA, based on data obtained from three wastewater treatment plants (WWTPs) in Catalonia, Spain, over a year (April 2015–May 2016). We also report our analysis of RVA in animal samples (porcine and bovine) collected at three local farms, in the same area of the WWTP sample collection, to determinate whether these animals may contribute as a source of contamination for the local population. Due to the role of human adenovirus (HAdV) as an indicator of human fecal contamination in water matrices, HAdV was considered as an alternative internal control over the studied samples.

## 2. Material and Methods

### 2.1. Raw Sewage Samples

Raw sewage samples were collected over a 1-year period between April 2015 and May 2016, from three WWTPs located in Catalonia, Spain. Among them was EDAR Sant Adrià de Besòs, with a capacity of 525,000 m^3^/day and which receives sewage from more than half the population of the Barcelona metropolitan area in the municipalities of Badalona, Barcelona (three quarters), Montgat, Sant Adrià de Besòs, Santa Coloma de Gramenet, Tiana and part of Montcada and Reixac, comprising around 2,800,000 people. Another source was EDAR Baix Llobregat, with a capacity of 315,000 m^3^/day, which receives sewage from Barcelona (one quarter), Cornellà de Llobregat, El Prat de Llobregat, Esplugues de Llobregat, L’Hospitalet de Llobregat, Sant Joan Despi, Sant Boi de Llobregat (partially), Santa Coloma de Cervelló and Saint Just Desvern (partially), equivalent to more than 1,700,000 inhabitants.

The remaining plant was EDAR Granollers (Vallès Oriental), located in the town of Granollers, with a capacity of 30,000 m^3^/day and which receives sewage from the municipalities of Canovelles, Les Franqueses of the Vallès and Granollers, with a population of 112,154 inhabitants. A total of 20 raw sewage samples were collected over the four annual seasons. At each sampling point, a 42 mL raw sewage sample was collected in sterile plastic bottles, kept at 4 °C and transported to the laboratory for immediate analysis.

### 2.2. Animal Fecal Samples

In order to estimate the patterns of circulation of RVA in animals in the region, we collected 1 fecal sample from a 7-week-old diarrheic piglet and 8 samples pools (3 samples in each pool) collected from the floor of pens of diarrheic and non-diarrheic pig and cattle of different ages, in the city of Vic, Catalonia. To note, this city is known for its production of sausages and other pork derivatives. In all visited farms, diarrheal animals are kept separately from healthy animals. The samples were collected in 50 mL sterile plastic bottles, kept at 4 °C and transported to the laboratory for immediate analysis.

### 2.3. Virus Concentration

The samples were concentrated using the ultracentrifugation method as previously described [14]. To avoid false negative results and to evaluate the presence of inhibitors, each sample was inoculated with 250 µL of bacteriophage MS2 (1 × 10^6^ gene copies—GC) as an internal control before the concentration assay and detected by Taqman^®^ RT-qPCR for viral recovery analysis [15].

### 2.4. Nucleic Acid Extraction, Reverse Transcription (RT) and Quantitative PCR (qPCR)

Viral nucleic acid was extracted using the commercial QIAmp Viral RNA kit^®^ (Qiagen Inc., Hilden, Germany). The RT-qPCR was performed for detection and quantification of RVA as previously described [16]. A standard curve (5 × 10^0^–5 × 10^7^ RT-PCR Units (RT-PCR U)/per reaction) was generated using 10-fold serial dilutions of a 473 bp qBlock^®^ gene fragment specific for the NSP3 gene from RVA (IDT—Integrated DNA Technologies, Coralville, IA, USA). The complementary DNA (cDNA) was obtained for RVA genotyping analysis using pd(N)6 (Random Hexamer, Amersham Biosiences) and SuperScript^®^ III as Reverse Transcriptase (Invitrogen, Carlsbad, CA, USA). HAdV was analyzed by qPCR as described by [17] and was considered as a second internal process control in WWTPs samples, as is considered as an indicator of human fecal contamination in water matrices. The limits of detection (LOD) for RVA and HAdV were 5.29 × 10^4^ RT-PCR U/L and 5.10 × 10^3^ GC/L, respectively.

### 2.5. VP4, VP6 and VP7 Amplification

Complementary DNA (cDNA) product was used as the template for amplification of the VP6, VP7 and VP8* portion of the VP4 gene segments. RT-PCR was performed using the primers VP6F and VP6R [18], 9Con1/9Con2 [19] and 4Con3/4Con2 [20], to the amplification of genes VP6, VP7 and VP8*, respectively. G (VP7) and P (VP8*) genotyping methods were conducted according to protocols established by the World Health Organization (WHO), available at http://apps.who.int/iris/bitstream/10665/70122/1/WHO_IVB_08.17_eng.pdf.

### 2.6. Sequencing of the VP6 RT-PCR Products

The 379 bp VP6 amplicons (nt 747–1126) obtained from RVA-positive strains were purified using a DNA Cleaner and Concentrator kit (D4013, Zymo Research Corp., Irvine, CA, USA), following the manufacturer’s recommendations. The purified DNA amplicons were subjected to a sequencing reaction using the Big Dye Terminator Cycle Sequencing Kit version 3.1 (Applied Biosystems, Foster City, CA, USA) and the primers VP6F and VP6R at 3.2 pmol. The BioEdit Sequence Alignment Editor Program^®^ was used to edit and align the sequences obtained [21]. Genetic analysis was performed using the neighbor-joining method with 2000 bootstrap replicates and the genetic distance was calculated using Kimura’s two-parameter matrix. A phylogenetic tree was constructed using MEGA v.7 software [22].

### 2.7. Statistical Analysis

All data were statistically analyzed using R software^®^ 3.5.1 [23]. First, descriptive statistics of all the quantifications and recoveries were obtained. Additionally, the Kruskal–Wallis non-parametric test was used to evaluate the differences in the RVA and HAdV concentrations in raw sewage between seasons. Post-hoc pairwise comparisons after the Kruskal–Wallis test were performed using the Tukey and Kramer (Nemenyi) test with Tukey-Dist approximation for independent samples with the PMCMR package in R [24]. Finally, Spearman’s rank correlation coefficient was used to evaluate the quantifications observed for HAdV and RVA.

## 3. Results and Discussion

RVA and HAdV genome concentrations were assessed over a one-year study in raw sewage samples from three WWTPs from the region of Catalonia, Spain. RVA was detected in 18 out of 20 sewage samples (90%) by RT-qPCR, with a mean viral load ranging from 3.96 × 10^4^ to 3.30 × 10^8^ RT-PCR U/L over the studied period (Table 1). The presence of RVA in a large portion of samples indicates the high circulation of this virus in Catalonia. Both high- and low-income countries have indicated the presence of RVA in WWTP samples, thus demonstrating the great dispersion of this virus in the environment worldwide [25,26,27].

All samples were found positive for the bacteriophage MS2 (our internal control), with recovery efficiency ranging from 9.8% to 88.2%. This variation may be justified by the complex nature of the raw sewage samples. Previous studies have shown important variation rates in viral recovery between concentration methods [28,29,30] and once RVA and MS2 do not have the same characteristics (dsRNA vs. bacteriophage ssRNA), the efficiency of recovery for both viruses is not expected to be correlated.

**Figure 1:**
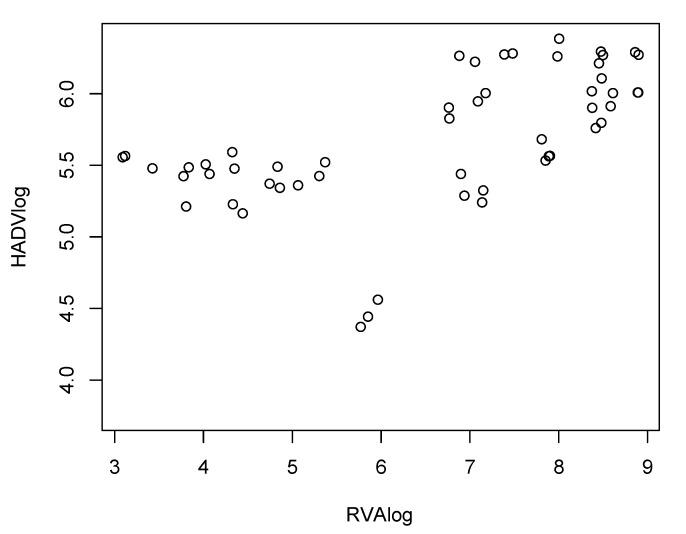
Correlation analysis between human adenovirus (HAdV) and rotavirus A (RVA) circulation in Catalonia. HAdV and RVA were detected and viral loads were estimated through qPCR (HAdV) and RT-qPCR (RVA) analysis of 42 mL raw sewage samples. The correlation analysis was performed using R software package.

**Figure 2:**
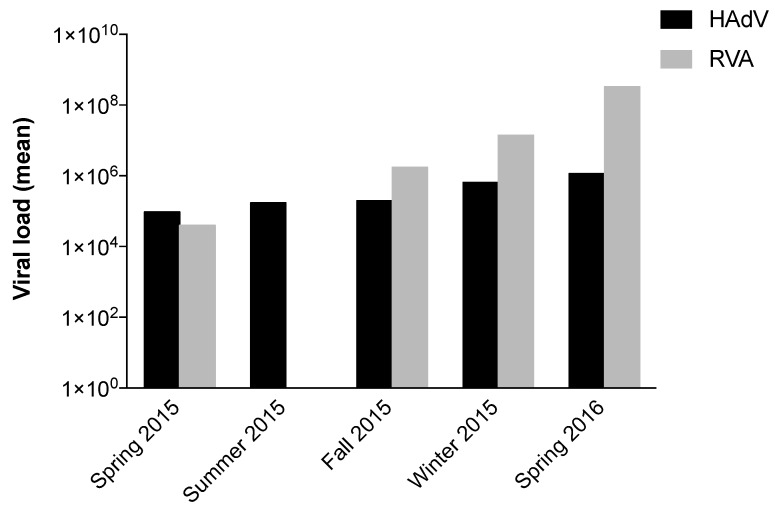
Human adenovirus (HAdV) and rotavirus A (RVA) circulation in raw sewage samples by season. Seasonal distribution of HAdV and RVA detected by qPCR and RT-qPCR, respectively, in raw sewage samples collected between spring 2015 and spring 2016 in Catalonia.

The concentrations of HAdV and RVA varied significantly between different seasons (*p*-value < 0.000 in both viruses). Both viruses presented a similar pattern of circulation over the seasons. with significant *p*-values comparing viral detection in winter and spring with that in summer and autumn (Table 2); this suggests greater viral detection in the colder months in the northern hemisphere for both viruses, with a moderate correlation (*R* = 67.2%, *p*-value < 0.000) between viral detection and seasonality (Figure 1). In accordance with data on RVA seasonality in Europe, this virus was not detected in the months of June and July (summer) in Barcelona (Figure 2), corroborating previous studies that have shown a large circulation of RVA in the cooler months in temperate countries [31,32]. It is important to emphasize that lack of detection of the virus does not imply that there was no RVA circulation in this period; instead, viral detection was below the LOD, due to the low sensitivity of RT-qPCR for RVA detection.

The VP4 gene was successfully characterized in all samples collected in 2015, showing the circulation of the genotypes P[4], P[8], P[9] and P[10] in Barcelona. The VP7 gene could only be genotyped in samples collected in April 2016 from Besòs and Granollers, showing the circulation of genotypes G2, G3, G9 and G12 in raw sewage from Barcelona (Table 3). These results corroborate a previous study [33], which showed the circulation of the genotypes G2, G3, G9, P[4], P[8] and P[9] in Barcelona; more specifically, at the Sant Adrià de Besòs WWTP. A study conducted between 2010 and 2011 [26] also showed RVA circulation in urban wastewater samples from different Italian cities. Corroborating our present data, those authors detected a large variety of G and P genotypes, such as G1, G2, G3, G4, G6, G9, G26, P[4], P[6], P[8], P[9], P[14] and P[19], as well as combined genotypes: G1+G2-P[4]+P[8].

The G12 genotype was detected at the Granollers WWTP in April 2016. This genotype, previously considered unusual, has been reported in association with cases of hospitalization in several studies worldwide, as it is the 6th most prevalent human RVA genotype [34]. The G12 strain detected in this study was sequenced and the result showed a high degree of nucleotide similarity with other human wild-type G12 strains circulating recently in different continents: Africa, America and Europe.

**Figure 3:**
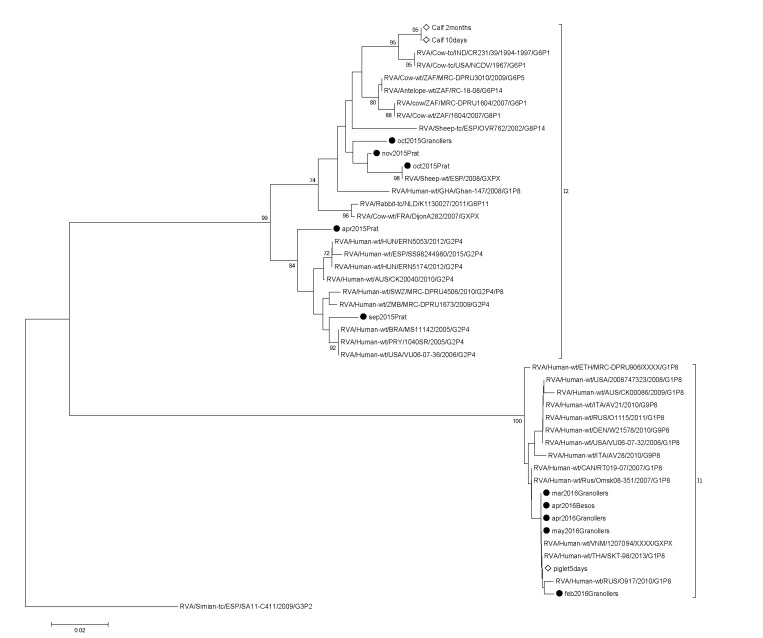
Phylogenetic analysis of the rotavirus A (RVA) VP6 gene from raw sewage and animal samples. The raw sewage samples from the present study are indicated with a black dot followed by month, year and wastewater treatment plant of collection. Samples recovered from animals are indicated with white diamonds.

RVA-positive strains, by the qPCR method, were subjected to VP6 amplification and Sanger sequencing. The results showed the circulation of the genotypes I1 and I2 in Catalonia (Figure 3). Samples collected in 2015 belonged to the I2 genotype and were grouped in a different cluster together with human and animal (antelope, cow and sheep) wild-type strains. On the other hand, samples collected in 2016 belonged to the I1 genotype, grouped together with human wild-type strains of different G/P genotypes circulating worldwide.

The analysis conducted on animal pool samples showed a high level of RVA circulation in different farms (see Supplementary Materials). Seven out of the nine samples (78%) were positive for RVA, either via RT-qPCR or RT-PCR methods. Samples recovered from animals under seven months of age showed positivity for RVA with viral loads ranging from 3.11 × 10^5^ to 7.60 × 10^8^. The two remaining pool samples were recovered from 7-month-old piglets and 8-month-old calves; thus, our results reinforce previous findings in the literature that describe RVA as one of the most common viruses detected in younger animals (1–8 weeks of age) [35,36]. Despite the small number of animals enrolled in our study, the differences concerning in RVA circulating in raw sewage and animals from the studied area, suggest that RVA detected in WWTPs may have a distinct origin than the surveyed animals.

It is important to highlight that the detection of high or low levels of virus in a specific geographic area depends not only on virus excretion levels, but also on the protocol used for detection [37]. We observed that there is no statistical significance between PCR and qPCR results for RVA detection, although previous studies showed greater sensitivity of qPCR in comparison to PCR [25,38]. The VP6 sequencing protocol was previously suggested to be more sensitive for RVA detection in environmental samples than amplification of the VP4 and VP7 genes [39].

The present study showed the high circulation of RVA in sewage samples from Catalonia, suggesting continuous RVA dissemination by individuals, allowing its detection despite the high dilution in sewage. Our results also highlight the importance of RVA surveillance studies in Spain. In our samples, RVA was detected in high concentrations in sewage samples, reflecting the high circulation of the virus in the population, although asymptomatically in most cases. Knowledge of RVA genotype circulation in the environment may help clinical monitoring of RVA infections, predicting which genotypes could be detected in clinical samples during RVA outbreaks.

Despite the relevance to environmental virology regarding RVA spreading in Catalonia in high concentrations, the small number of samples, the presence of possible inhibitors in sewage samples and the variable viral recovery values represent the main limitations of this study. The present data show RVA circulation in young animals (piglets and calves), though the sequences recovered from animal samples were not related to those found in sewage samples. Nevertheless, more studies are necessary to exclude the possibility of RVA infection through the consumption of raw or poorly cooked meat from infected animals.

## Figures and Tables

**Table 1:** Human adenovirus (HAdV) and rotavirus A (RVA) mean viral load during the study period. Quantification analysis of viral load (mean) detected by qPCR (HAdV) and RT-qPCR (RVA) in raw sewage samples collected between spring 2015 and spring 2016 in Catalonia.

Period	HAdV (CG/L)	RVA (RT-PCR U/L)
Spring 2015	9.51 × 10^4^	3.96 × 10^4^
Summer 2015	1.71 × 10^5^	-
Fall 2015	1.98 × 10^5^	1.73 × 10^6^
Winter 2015	6.50 × 10^5^	1.41 × 10^7^
Spring 2016	1.16 × 10^6^	3.30 × 10^8^

**Table 2:** Pairwise comparison analysis (*p*-values) between viral load and season using the Tukey and Kramer (Nemenyi) test.

HAdV				RVA		
	Summer	Fall	Winter	Summer	Fall	Winter
**Fall**	0.97538			0.0743		
**Winter**	0.00077	2.0 × 10^−8^		3.6 × 10^−^^6^	0.0011	
**Spring**	0.00941	1.6 × 10^−5^	0.93618	1.3 × 10^−^^8^	2.8 × 10^−6^	0.4376

**Table 3:** VP4, VP7 and VP6 rotavirus A (RVA) genotypes during the study period. RVA genotypes circulating in Catalonia detected by qualitative RT-PCR and/or Sanger sequencing. The empty spaces in the table indicate that it was not possible to genotype the RVA strains from the given season.

Season	RVA Genotypes		
	VP4	VP7	VP6
**Spring 2015**	P[4]		I2
**Summer 2015**			
**Fall 2015**	P[4], P[8], P[9], P[10]		I2
**Winter 2015**	P[4], P[8], P[9]		I1
**Spring 2016**	P[4], P[8]	G2, G3, G9, G12	I1

## Supplementary Materials

Supplementary materials can be found at https://www.mdpi.com/1999-4915/12/3/318/s1, Table S1: Data on mean RVA load detected from fecal samples recovered from animals.
