# Supplementary material for: High Prevalence of Rotavirus A in Raw Sewage Samples from Northeast Spain
Source: Viruses. 2020 Mar 16;12(3):318. doi: 10.3390/v12030318 (PMC7150846; doi:10.3390/v12030318)
Supplement: Supplementary file 1 [file viruses-12-00318-s001.pdf]

Sample	Age	mean RVA load (RT-PCR U/L)
Pool calf	8 months	neg
Pool piglet	5 days	1,08E+06
Diarrheic piglet	7 weeks	7,52E+07
Pool piglets	7 months	neg
Pool piglets	7 weeks	1,29E+07
Pool piglets	15 weeks	3,41E+06
Pool diarrheic piglets	5 days	3,11E+05
Pool calf	2 months	7,60E+08
Pool calf	10 days	6,76E+07
